# Gut microbiota-derived indole 3-propionic acid partially activates aryl hydrocarbon receptor to promote macrophage phagocytosis and attenuate septic injury

**DOI:** 10.3389/fcimb.2022.1015386

**Published:** 2022-10-10

**Authors:** Zhi-Bin Huang, Zhen Hu, Chen-Xin Lu, Si-Dan Luo, Yu Chen, Zhi-Peng Zhou, Jing-Juan Hu, Fang-Ling Zhang, Fan Deng, Ke-Xuan Liu

**Affiliations:** ^1^ Department of Anesthesiology, Nanfang Hospital, Southern Medical University, Guangzhou, China; ^2^ Department of Anesthesiology, Shengli Clinical Medical College of Fujian Medical University, Fujian Provincial Hospital, Fuzhou, China; ^3^ Department of Anesthesiology, Fuzhou Second Hospital, Fuzhou, China; ^4^ Department of Urology, Nanfang Hospital, Southern Medical University, Guangzhou, China

**Keywords:** gut microbiota, macrophage, phagocytosis, sepsis, infection, indole 3-propionic acid

## Abstract

Sepsis is associated with a high risk of death, and the crosstalk between gut microbiota and sepsis is gradually revealed. Indole 3-propionic acid (IPA) is a gut microbiota-derived metabolite that exerts immune regulation and organ protective effects. However, the role of IPA in sepsis is not clear. In this study, the role of IPA in sepsis-related survival, clinical scores, bacterial burden, and organ injury was assessed in a murine model of cecal ligation and puncture-induced polymicrobial sepsis. Aryl hydrocarbon receptor (AhR) highly specific inhibitor (CH223191) was used to observe the role of AhR in the protection of IPA against sepsis. The effects of IPA on bacterial phagocytosis by macrophages were investigated *in vivo* and vitro. The levels of IPA in feces were measured and analyzed in human sepsis patients and patient controls. First, we found that gut microbiota-derived IPA was associated with the survival of septic mice. Then, in animal model, IPA administration protected against sepsis-related mortality and alleviated sepsis-induced bacterial burden and organ injury, which was blunted by AhR inhibitor. Next, *in vivo* and vitro, IPA enhanced the macrophage phagocytosis through AhR. Depletion of macrophages reversed the protective effects of IPA on sepsis. Finally, on the day of ICU admission (day 0), septic patients had significantly lower IPA level in feces than patient controls. Also, septic patients with bacteremia had significantly lower IPA levels in feces compared with those with non-bacteremia. Furthermore, in septic patients, reduced IPA was associated with worse clinical outcomes, and IPA in feces had similar prediction ability of 28-day mortality with SOFA score, and increased the predictive ability of SOFA score. These findings indicate that gut microbiota-derived IPA can protect against sepsis through host control of infection by promoting macrophages phagocytosis and suggest that IPA may be a new strategy for sepsis treatment.

## Introduction

Sepsis is a complex and highly heterogeneous clinical syndrome characterized by an unbalanced response of the host to infection and is associated with acute organ dysfunction and a high risk of death (Cecconi et al., 2018). Worldwide, it is estimated that there are 31.5 million cases of sepsis and 19.4 million cases of severe sepsis each year, and about 5.3 million patients may die of sepsis (Fleischmann et al., 2016). In fact, the current treatments of sepsis are mainly anti-infection, fluid resuscitation and organs support (Yao et al., 2020). Therefore, in view of the high morbidity, high mortality and lack of effective treatment of sepsis, further understanding of the mechanism of the occurrence and development of sepsis and exploring effective treatment are urgent health problems that need to be solved. In recent years, a review has systematically revealed the crosstalk between gut microbiota and sepsis (Niu and Chen, 2021). However, the crosstalk between gut microbiota metabolites and sepsis remains poorly understood.

Sepsis induces significant gut dysbiosis (Agudelo-Ochoa et al., 2020), while gut microbiota can directly or indirectly affect the pathophysiological process of host intestinal and extraintestinal organs through bioactive metabolites (Deng et al., 2021; Deng et al., 2021; Hu et al., 2022). Indole 3-propionic acid (IPA) is a tryptophan deamination product derived from *Lactobacillus reuteri, Akkermansia and Clostiridum genus*, which mediate intracellular signaling activity (Dodd et al., 2017; Alexeev et al., 2018; Konopelski and Mogilnicka, 2022). In recent years, numerous studies have shown that IPA exerts organ protective effects, including improving intestinal barrier dysfunction, reducing inflammatory response and attenuating cognitive impairment caused by diabetes (Bendheim et al., 2002; Venkatesh et al., 2014; Rothhammer et al., 2016; Dodd et al., 2017; Liu et al., 2020; Xiao et al., 2020). In addition, gut microbiota-derived IPA can regulate systemic immunity (Dodd et al., 2017). Moreover, in the early stage of sepsis, the dominant pathogen can excessively activate the body’s innate immune system and produce excessive inflammatory response (Wiersinga et al., 2014). Interestingly, in other animal models, IPA down-regulates the expression of proinflammatory cytokines, including TNF-α, IL-1β and IL-6 (Zhao et al., 2019). However, whether gut microbiota metabolite IPA plays a part in alleviating sepsis and the underlying molecular mechanism are elusive.

Macrophages have two key roles: to respond quickly to infection and injury and to help repair tissue damage caused by this response (Wang et al., 2020; Liu et al., 2021). Studies have shown that severe sepsis is associated with progressive macrophages dysfunction (Huang et al., 2009; Hotchkiss et al., 2013; Delano and Ward, 2016; Rodriguez et al., 2019; Jin et al., 2020). Aryl hydrocarbon receptor (AhR), a ligand-activated transcription factor, regulates immune homeostasis in the process of infection (Gutiérrez-Vázquez and Quintana, 2018). AhR-KO mice exerted enhanced susceptibility to LPS treatment and hypersensitivity to septic shock, mainly due to macrophage dysfunction (Sekine et al., 2009; Zhu et al., 2018). The metabolites of tryptophan by gut microbiota are the physiological source of AhR agonist (Shinde and McGaha, 2018).

In this study, we investigated the role of IPA-induced AhR activation in controlling the infection during sepsis using the cecal ligation and puncture (CLP) model in mice. We also measured the IPA levels of feces in septic patients and analyzed the association between IPA and clinical phenotypes of sepsis.

## Materials and methods

### Human and animal ethics

This study of human subjects was approved by the Research Ethics Board of Fujian Provincial Hospital, Fuzhou, China (Approval number: K2021-02-005). All patients signed written informed consent. The care and handling of the animals were in accord with the National Institutes of Health guidelines and the study of animals was approved by Animal Care and Use Committee of Southern Medical University, Guangzhou, China.

### Human subjects

Stools from sepsis patients and inpatient non-sepsis controls were used in the present study. Sepsis was identified according to The Third International Consensus Definitions for Sepsis and Septic Shock (Sepsis-3) (Singer et al., 2016).

### ABX mice

Male C57BL/6 mice (6-to 8-week-old) received antibiotics (vancomycin, 100 mg/kg; neomycin sulfate 200 mg/kg; metronidazole 200 mg/kg; and ampicillin 200 mg/kg) intragastrically once a day for 5 days to deplete the gut microbiota.

### Cecal ligation and puncture

Male C57BL/6 mice (8-10-week-old) were purchased from Beijing HFK Bioscience Co., Ltd and raised at Nanfang Hospital of Southern Medical University. Polymicrobial sepsis was induced by cecal ligation and puncture as described in previous study (Rittirsch et al., 2009). All experimental procedures were in accordance with the National Institutes of Health guidelines and were approved by Animal Care and Use Committee of Southern Medical University.

### Administration of IPA *in vivo* and *vitro*

For pre-treatment, mice were treated with IPA (Sigma-Aldrich, #220027-1, Shanghai, China; 20 mg/kg, diluted with 200 μl PBS containing 0.5% DMSO) or vehicle (PBS containing 0.5% DMSO) by oral gavage for 5 consecutive days (once a day) before CLP. For post-treatment, mice were treated with IPA (20 mg/kg, diluted with 200 μl PBS containing 0.5% DMSO) or vehicle (PBS containing 0.5% DMSO) by oral gavage for 3 consecutive days (once a day) after CLP. *In vitro*, cell lines were treated with IPA (37.6 μg/ml) for 24h before phagocytic assay.

### Murine sepsis score

Murine sepsis score of septic mice was assessed at 6 h, 12 h and 24 h after CLP. The score of each animal was assessed as follows (points). [a] Appearance: smooth coat (0), piloerected hair (1), piloerected back (2), “puffy” with or without piloerection (3), emaciation with or without piloerection (4). [b] Level of consciousness: active (0), active but avoids standing upright (1), ambulant but noticeably slowed (2), moving only when provoked (3), remaining stationary when provoked (4). [c] Activity: normal amount of activity (0), only moving around bottom of cage (1), remaining stationary with occasional investigative movements (2), remaining stationary (3), experiencing tremors (4). [d] Response to stimulus: responding immediately to auditory stimulus or touch (0), slow or no response to auditory stimulus-strong response to touch (moves to escape) (1), no response to auditory stimulus-moderate response to touch (moves a few steps) (2), no response to auditory stimulus-mild response to touch (no locomotion) (3), no response to auditory stimulus-little or no response to touch or without righting itself if pushed over (4). [e] Eyes: fully open (0), not fully open, possibly with secretions (1), at least half closed, possibly with secretions (2), half closed or more, possibly with secretions (3), closed or milky (4). [f] Respiration rate: normal, rapid mouse respiration (0), slightly decreased respiration (rate not quantifiable by eye) (1), moderately reduced respiration (rate at the upper range of quantifying by eye) (2), severely reduced respiration (rate easily countable by eye, 0.5 s between breaths) (3), extremely reduced respiration (>1 s between breaths) (4). [g] Respiration quality: normal (0), brief periods of laboured breathing (1), 1aboured without gasping (2), laboured with intermittent gasps (3), gasping (4).

### Experimental group

*Sham + vehicle group*: mice were treated with oral gavage of vehicle (PBS containing 0.5% DMSO) for 5 consecutive days (once a day), and then sham. *Sham + IPA group*: mice were treated with oral gavage of IPA (20mg/kg, diluted with 200μl PBS containing 0.5% DMSO) for 5 consecutive days (once a day), and then sham. *CLP + vehicle group*: mice were treated with oral gavage of vehicle for 5 consecutive days, and then CLP. *CLP + IPA group*: mice were treated with oral gavage of IPA for 5 consecutive days, and then CLP. *IPA + CH223191 group*: mice were treated with oral gavage of IPA and intraperitoneal injection of CH223191(aryl hydrocarbon receptor inhibitor, 5 mg/kg) for 5 consecutive days (once a day) before CLP. *IPA + macrophages depletion group*: mice were treated with oral gavage of IPA for 5 consecutive days and intraperitoneal injection of 200 μl clodronate liposomes (LIPOSOMA, China, #CP-005-005) 3 days before CLP.

### Quantification of IPA

*Mice*: 8-10-week-old male C57BL/6 mice were acclimated for 7 days. Then, the stools were collected under SPF conditions after ABX treatment. 0.1g feces were diluted with 1ml saline. In brief, the feces soaked in normal saline for about 15 minutes, and then shaked and mixed well, and then centrifuged at 800rpm for 3 minutes. The supernatant was used to determine the level of IPA by enzyme-linked immunosorbent assay (ELISA) kit (Shanghai Jingkang Biotechnology Co., Ltd., China #JLC3144) or stored at -80°C. *Human*: Stool samples from patients were collected at sepsis onset. Dissolution and centrifugation were the same as described above. The level of IPA in supernatant was determined by ELISA kit (Xiamen Lunchangshuo Biotechnology Co., Ltd., China, # LCS14702).

### Cell culture

Human monocytic THP-1 cells were obtained from ATCC and maintained at 2×10^5^/ml in Roswell Park Memorial Institute medium (RPMI 1640, Invitrogen) containing 10% of heat inactivated fetal bovine serum (FBS, Invitrogen) and supplemented with 2 mmol/L L-glutamine. THP-1 monocytes were incubated in RPMI medium for 24h and differentiated into macrophages using 200 ng/mL phorbol 12-myristate 13-acetate (PMA, Sigma, P8139) for 3 days.

### Serum biochemistry

At 12 hours after CLP, mice were thoracotomized to fully expose the heart and blood was slowly drawn from the right ventricle with a 1ml syringe. The blood was placed in 4°C overnight and the serum was obtained by centrifugation (3500 rpm at 4°C) for 10 minutes and stored at -80°C. The levels of alanine aminotransferase (ALT), aspartate aminotransferase (AST), lactate dehydrogenase (LDH), creatine kinase (CK), and lactic acid (LAC) in serum were determined using IDEXX Catalyst One^®^ Automatic Biochemical Analyzer.

### Measurement of cytokines in serum and peritoneal lavage fluid

At 12 hours after CLP, the peritoneum was fully exposed, and 5 ml pre-cooled 1×PBS was injected into the abdominal cavity of mice. 1ml lavage fluid was obtained after gently rubbing the abdominal cavity, and then was stored at -80°C. Serum was collected as described above. The concentrations of IL-1β, TNF-α, and IL-6 in serum and peritoneal lavage fluid were determined using ELISA kits (Jiangsu Boshen Biotechnology Co., Ltd, China; Proteintech, USA) according to the manufacturer’s instructions.

### Measurement of colony forming units

Serum and peritoneal lavage fluid were collected as described above. Spleen homogenate was collected as follow: At 12 hours after CLP, the spleen of mice was removed aseptically, and then placed in a 1.5 ml sterile tube and fully cut it up with aseptic surgical scissors. Spleen fragments were fully mixed with 1ml aseptic 1×PBS, and the supernatant was obtained by 1000 rpm centrifugation for 5 minutes. Serum, peritoneal lavage fluid and spleen homogenate supernatant were diluted 25 times, 30 times and 30 times by 2% sterilized LB broth (Solarbio, China, #L8291), respectively, and then were incubated at 37°C with 200 rpm shaking for 24 hours. The count was calculated as CFUs per ml. McFarland (MCF) turbidity (1MCF corresponding to 3×10^8^ CFU/ml) was detected by bacterial turbidimeter.

### *In vitro* staining FITC-d-Lys into *E. coli*

*E. coli* was grown at 37°C in LB medium until OD600 reached 0.6. The medium was diluted to OD600 = 0.3 with fresh medium containing FITC-d-Lys (Xiamen Bioluminor Bio-Technology Co., Ltd., China; Final concentration: 0.1 mM, usually adding 5 μl of stock solution to per mL cell medium). The diluted bacteria were further incubated at 37°C until OD600 = 1.0-1.5. The bacteria were centrifuged, washed with LB medium three times, and then resuspended in sodium phosphate buffer (100 mM, pH 7.4) or cell culturing medium of interest.

### Phagocytosis measurement by using FACS

*In vitro.* THP-1 monocytes/macrophages (0.1×10^6^) were cultured in 6-well plates in DMEM containing 10% FBS. The next day, plates were washed, nonadherent cells were discarded, and DMEM containing 10% FBS, mixed with 0.1 × 10^8^ FITC-conjugated *E. coli* were added to macrophages for 30 minutes at 37°C to induce phagocytosis. Macrophages were washed twice with cold PBS and resuspended with 1×PBS containing 5 mM EDTA before staining.

*In vivo.* FITC-conjugated *E. coli* (10 × 10^7^) were injected into the peritoneal cavity of mice pretreated with vehicle, IPA with or without CH223191. Mice were sacrificed 30 minutes after injection. Peritoneal cells were stained and analyzed by Flexible Account Configuration System (FACS).

*FACS analysis.* After phagocytosis, THP-1 monocytes/macrophages and peritoneal macrophages were stained with fluorochrome-conjugated monoclonal antibody against F4/80-PE for 30 min at 4°C. Following incubation, all cells were lysed and washed 3 times with a FACS buffer. Fluorescent cells were detected using a BD LSR Fortessa instrument with Diva software (BD Biosciences) and analyzed using FlowJo software (Tree Star Inc.).

### Histological score

Histological score was assessed as described in previous study (Suzuki et al., 1993; Ozdulger et al., 2003). In brief, hematoxylin and eosin-stained slides were prepared by using standard methods. Light microscopic analysis of lung and liver was performed by blinded observation. For lung, the results were classified into four grades, where grade 1 represented normal histopathology; grade 2 indicated only few neutrophil leukocyte infiltration; grade 3 represented moderate neutrophil leukocyte infiltration perivascular edema formation, and partial destruction of pulmonary architecture; and finally grade 4 included dense neutrophil leukocyte infiltration, abscess formation, and complete destruction of pulmonary architecture. For liver, histological changes were scored from 0 to 4 based on the degree of cytoplasmic vacuolization, sinusoidal congestion, and necrosis of parenchymal cells.

### Statistics

Results were expressed as identified in legends. In brief, for comparing 2 groups, statistical tests included unpaired t test or 2-tailed nonparametric Mann-Whitney test. For comparing 3 or more groups, one-way ANOVA followed by Tukey’s Multiple Comparison Test was performed. *P* values of 0.05 or less were considered to denote significance. Correlations were tested by Spearman’s rank correlation test. For survival studies, Kaplan–Meier analyses followed by log-rank tests were performed. All analyses were done using GraphPad Prism version 8.0.1 (GraphPad Software, San Diego, CA) and MedCalc software version 19.2.

## Results

### Gut microbiota-derived IPA was associated with the survival of septic mice

**Figure 1:**
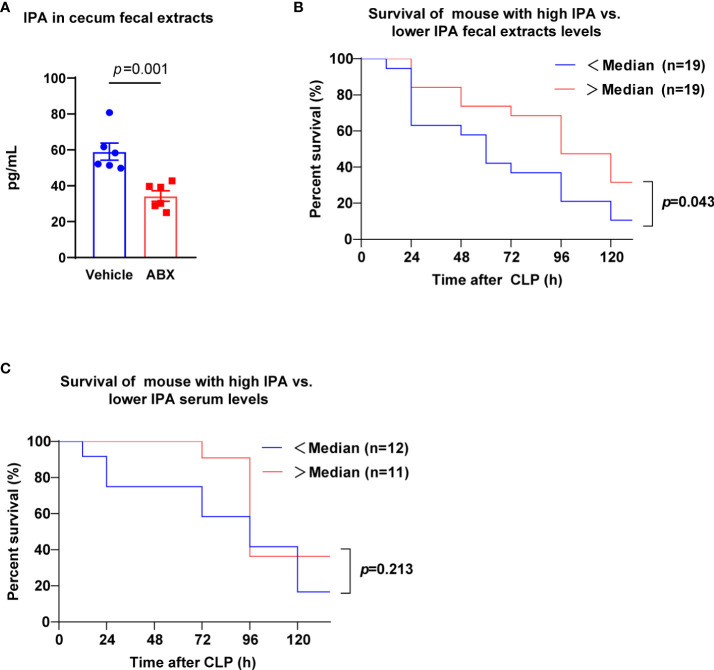
Gut microbiota-derived IPA was positively correlated with survival in septic mice. **(A)** The concentration of IPA in feces from mice was measured at the end of receiving 5 days of ABX. Data was shown as mean ± SEM; n=6; Mann Whitney U test. **(B)** Kaplan-Meier analysis (5 days after CLP) showing the survival of mice with high IPA fecal extracts levels (> ˃median, measured before CLP) versus the survival of mice with lower IPA (<median). n=19 per group; Log-rank (Mantel-Cox) test. **(C)** Kaplan-Meier analysis showing the survival of mice with high IPA serum levels versus lower IPA serum levels. n=11-12; Log-rank (Mantel-Cox) test. IPA, indole 3-propionic acid; ABX, antibiotics; CLP, cecal ligation and puncture.

Previously, it was showed that gut microbiota were found to produce IPA (Konopelski and Mogilnicka, 2022). Thus, we tested intestinal microbial metabolite IPA from mouse fecal extracts of mice treated with antibiotics (ABX) for 5 days. Notably, ABX treatment lessened the content of IPA in mice feces (**Figure 1A**). IPA was found to have potential anti-inflammatory and antioxidant properties (Negatu et al., 2020). Therefore, we explored whether the level of IPA in the feces before CLP was related to the survival of septic mice. Mice were divided into high-level group (more than or equal to the median) and low-level group (less than the median) by the interquartile range of feces IPA values. After lethal CLP, we observed the survival of mice for 5 days, the mice survival rate was decreased by 10.5% in IPA low-level group, but it was 26.3% in IPA high-level group (**Figure 1B**). According to the methods above, mice were divided into high-level group and low-level group by serum IPA values. The mice survival rate was 45.5% at 3 days and 16.7% at 5 days in serum IPA low-level group, but it was 91.7% and 36.4% in serum IPA high-level group (**Figure 1C**). Thus, these observations suggest that IPA derived from gut microbiota before CLP may have a potential protective effect on sepsis in mice.

### IPA pre-treatment protected against CLP-induced mortality and improved clinical scores in mice

**Figure 2:**
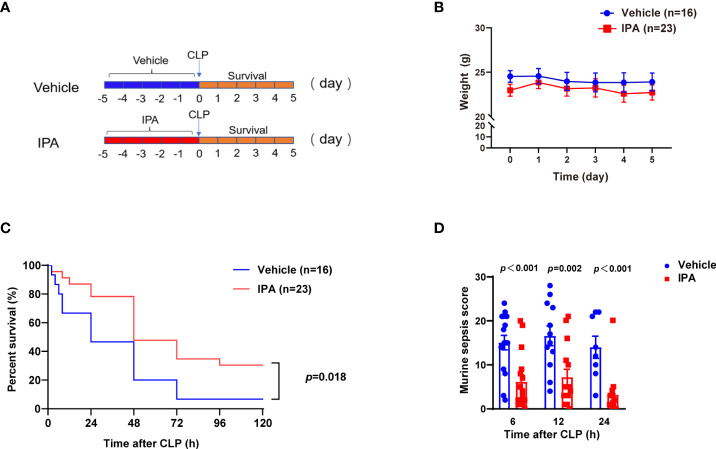
Effect of IPA on CLP-induced mortality and sepsis score in mice. **(A)** The study schedule. Oral gavage of IPA (20 mg/kg) was performed daily for 5 days before CLP. Survival was recorded for 5 days after CLP. **(B)** Body weights were compared among mice after oral gavage IPA or vehicle; Significant differences between two cohorts are performed by t tests. **(C)** Kaplan-Meier analysis of septic mice pre-treated with oral gavage IPA or vehicle; Log-rank test between IPA (n=23) and Vehicle (n=16) groups. **(D)** Murine sepsis score of septic mice at 6 h, 12 h and 24 h after CLP between IPA and vehicle groups; Significant differences between two cohorts are performed by t tests. IPA, indole 3-propionic acid; CLP, cecal ligation and puncture; n.s., no significance.

In view of the relationship between IPA and the survival of septic mice, we determined whether IPA was sufficient to improve mortality in lethal sepsis. Oral gavage of IPA (20 mg/kg) or vehicle was performed daily for 5 days before lethal CLP in mice, and the survival was recorded for 5 days after CLP (**Figure 2A**). IPA treatment had no significant effect on the body weight of mice (**Figure 2B**). The mice survival rate was decreased by 6.3% in vehicle group, but it was 30.4% in IPA group (**Figure 2C**). Compared with vehicle mice, IPA mice were protected against sepsis, as seen in their lower mortality rates, and had better clinical scores (**Figure 2D**). These results indicate that IPA has a protection effect against experimental sepsis.

### IPA pre-treatment alleviated lung and liver injury and inflammatory response in septic mice

**Figure 3:**
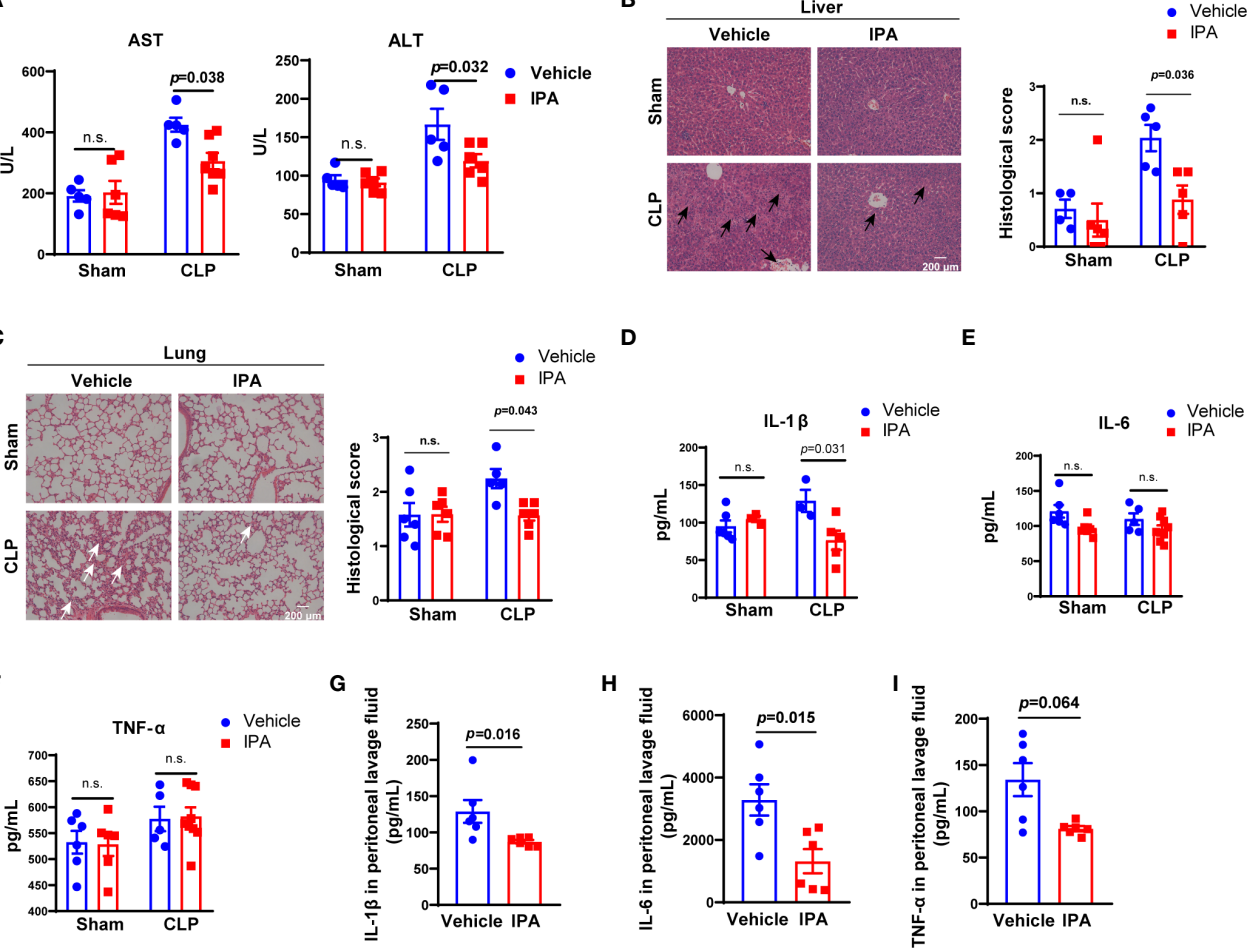
Effect of IPA on CLP–induced liver and lung injury and inflammatory response in mice. **(A)** Serological markers of organ injury including ALT and AST in serum of septic mice pre-treated with or without IPA (oral gavage, 20 mg/kg daily for 5 days) at 12 h after CLP. n=3-8, one-way ANOVA, multiple comparisons using Tukey’s multiple comparisons test. **(B, C)** Representative examples and histological scores of hematoxylins and eosin **(H&E)**–stained lung and liver tissues from mice pre-treated with or without IPA (n=4-6) at 12 h after lethal CLP. n=4-6, one-way ANOVA, multiple comparisons using Tukey’s multiple comparisons test. The black arrows point to the cytoplasmic vacuolization, sinusoidal congestion and necrosis of parenchymal cells in liver. The white arrows point to the neutrophil leukocyte infiltration, perivascular edema formation and destruction of pulmonary architecture of lung. **(D–F)** Concentration of the inflammatory cytokines IL-1β, IL -6, and TNF-αin serum at 12 h after CLP in septic mice pre-treated with or without IPA. n=3-9, one-way ANOVA, multiple comparisons using Tukey’s multiple comparisons test. **(G–I)** IL-1β, IL-6, TNF-α in peritoneal lavage fluid of septic mice at 12 h after CLP between IPA and vehicle groups; Mann Whitney U test. Data was shown as mean ± SEM. IPA, indole 3-propionic acid; CLP, cecal ligation and puncture, n.s., no significance.

The mortality of sepsis is caused by multiple organ failure caused by uncontrolled proliferation and spread of bacteria and excessive production of proinflammatory cytokines (Tom van der Poll et al., 2017). Accordingly, biochemical markers of liver were detected. Alanine aminotransferase (ALT) and aspartate aminotransferase (AST) in serum were elevated at 12 h after CLP induction in mice, and were lower in IPA pre-treatment mice than vehicle mice (**Figure 3A**). Likewise, administration of IPA alleviated the neutrophil leukocyte infiltration, perivascular edema formation and destruction of pulmonary architecture of lung in septic mice, and the degree of cytoplasmic vacuolization, sinusoidal congestion and necrosis of parenchymal cells in liver, which were reflected by lower pathology scores compared with vehicle-treated mice (**Figures 3B, C**). Serum concentrations of cytokines, such as IL-1β, IL-6 and TNF-α, were detected. At 12 h after CLP, the level of IL-1β was markedly lower in IPA pre-treatment mice than vehicle mice (**Figure 3D**). However, we observed no difference in the levels of IL-6 and TNF-α in serum between IPA and vehicle group (**Figures 3E, F**). We also examined inflammatory cytokines in peritoneal lavage fluid after 12 h in CLP mice, showing reduced levels of IL-1β, IL-6 and TNF-α in IPA pre-treated mice (**Figures 3G–I**). These results indicate that IPA replenishment plays a key role in protection against sepsis-induced vital organs injury and alleviates inflammatory response in different degrees in mice.

### AhR mediated the protective effects of IPA on septic mice

**Figure 4:**
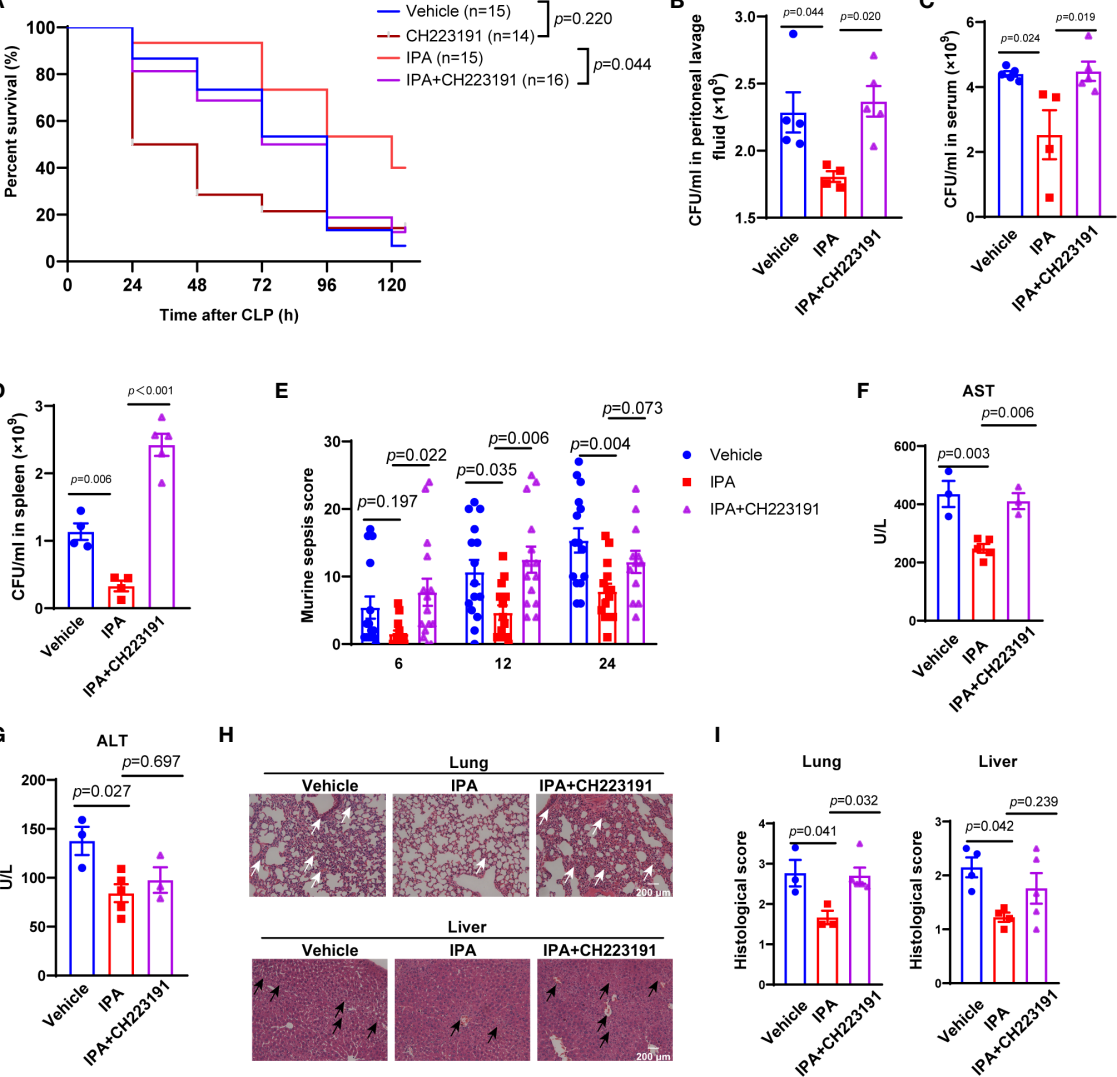
AhR was an important mediator in the protective effects of pre-treatment with IPA on septic mice. **(A)** Kaplan-Meier analysis of septic mice pre-treated with vehicle, CH223191, IPA (oral gavage, 20 mg/kg) with or without CH223191 (AhR inhibitor, intraperitoneal injection, 5 mg/kg) daily for 5 days. The survival rate was followed for 5 days after CLP; Log-rank. **(B–D)** Bacterial load in peritoneal lavage fluid **(B)**, blood **(C)**, or spleens **(D)** from mice pre-treated IPA (20 mg/kg daily for 5 days) with or without CH223191 at 12 h after CLP. n=4-5, one-way ANOVA, multiple comparisons using Tukey’s multiple comparisons test. **(E)** Murine sepsis score of septic mice at 6 h, 12 h and 24 h after CLP among vehicle, IPA and IPA+CH223191 groups, n=10-15, significant differences between two cohorts are performed by t tests. **(F, G)** Serological markers of organ injury including alanine aminotransferase (ALT) and aspartate aminotransferase (AST) in serum of septic mice pre-treated with IPA (oral gavage, 20 mg/kg daily for 5 days) with or without CH223191 (intraperitoneal injection, 5 mg/kg daily for 5 days) at 12 h after CLP. n=4-5, one-way ANOVA, multiple comparisons using Tukey’s multiple comparisons test. **(H, I)** Representative examples and histological scores of hematoxylins and eosin (H&E)–stained lung and liver tissues from septic mice pre-treated with IPA with or without CH223191 at 12 h after CLP. n=3-6, one-way ANOVA, multiple comparisons using Tukey’s multiple comparisons test. The black arrows point to the cytoplasmic vacuolization, sinusoidal congestion and necrosis of parenchymal cells in liver. The white arrows point to the neutrophil leukocyte infiltration, perivascular edema formation and destruction of pulmonary architecture of lung. Data was shown as mean ± SEM **(B-D)**. IPA, indole 3-propionic acid; CLP, cecal ligation and puncture, n.s., no significance.

Aryl hydrocarbon receptor (AhR), activated by the metabolites of gut microbiota, mediates the dysfunction of macrophages in lipopolysaccharide-induced septic mice (Sekine et al., 2009; Gutiérrez-Vázquez and Quintana, 2018). Thus, we aimed to elucidate whether IPA played a protective role in sepsis was related to AhR. We found that treatment with CH223191 (AhR inhibitor) significantly eliminated the effect of IPA on the survival of septic mice (**Figure 4A**). Same effects were observed in the bacterial load of serum, peritoneal lavage fluid and spleen from mice at 12 h after CLP (**Figures 4B–D**). The score reflecting the severity of sepsis also showed the same effect at 6 h, 12 h and 24 h after CLP in mice (**Figure 4E**).

Activation of AhR mediates cytoprotective effects in vital organs (Volkova et al., 2011; Yan et al., 2019). At 12 h after CLP, serum concentrations of AST, but not ALT, was significantly decreased in mice exposed to IPA compared with vehicle, but mice treated with IPA and CH223191 weakened the reduction (**Figures 4F, G**). Further analysis showed that at 12 h after CLP, CH223191 treatment blunted the organ protective effects of IPA on septic mice, which was associated with marked damage and inflammatory cell infiltration in the lung, and more obvious necrosis and bleeding areas in the liver (**Figures 4H, I**). Interestingly, post-treatment with IPA had no effect on CLP-induced mortality in mice (**Supplemental Figures 1A, B**). Taken together, AhR is involved in the protective effects of IPA on septic mice.

### IPA enhanced macrophages phagocytosis *via* AhR

**Figure 5:**
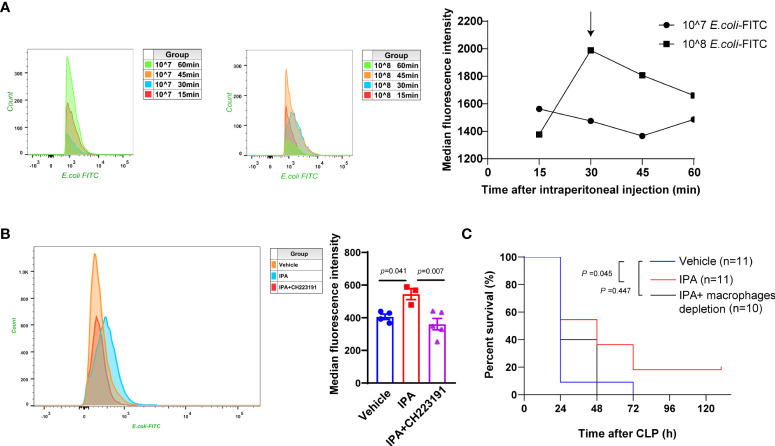
Enhanced bacterial phagocytosis by macrophages *via* AhR mediated the survival protective effect of IPA on septic mice. **(A)** *In vivo*, flow cytometric analysis of peritoneal macrophage phagocytosis of FITC-labeled *E. coli* (10×10^7^ or 10×10^8^) intraperitoneally injected into wild type mice at 15 min, 30 min, 45 min, and 60 min after injection. The line chart representing the mean fluorescence intensity of macrophages after phagocytosis. **(B)** *In vivo*, flow cytometric analysis of peritoneal macrophage phagocytosis of FITC-labeled *E. coli* (10×10^7^) intraperitoneally injected into mice treated with IPA (oral gavage, 20 mg/kg daily for 5 days) with or without CH223191 (intraperitoneal injection, 5 mg/kg daily for 5 days) at 30min after injection. n=3-5, one-way ANOVA, multiple comparisons using Tukey’s multiple comparisons test. **(C)** Kaplan-Meier analysis of septic mice pre-treated with oral gavage vehicle, IPA (20 mg/kg) with or without clodronate liposomes (depletion of macrophages, intraperitoneal injection, 100 μl/10 g). The survival was followed for 5 days after CLP; Log-rank. Data was shown as mean ± SEM **(B)**. *E.coli, Escherichia coli*; IPA, indole 3-propionic acid; CLP, cecal ligation and puncture.

We have shown above that IPA decreased the bacterial load in serum, peritoneal lavage fluid and spleen of septic mice. These indicate that IPA may play a detrimental role in sepsis by preventing the bacterial dissemination. Studies have shown that the elimination of bacteria by a macrophage-dependent manner affected the survival and organ damage in septic mice (Hotchkiss et al., 2013; Delano and Ward, 2016; Yang et al., 2019). We next assessed whether IPA enhanced the phagocytosis of E. coli by macrophages. The mean fluorescence intensity (MFI) of peritoneal macrophages in mice injected with 10×10^8^ FITC-labeled E. coli bacteria were generally higher compared with those injected with 10×10^7^ FITC-labeled E. coli bacteria. With the increase of phagocytosis time, the peak of MFI in peritoneal macrophages appeared at 30 min after mice injected with 10×10^8^ FITC-labeled E. coli bacteria (**Figure 5A**). Thus, mice intraperitoneally injected with 10×10^8^ FITC-labeled E. coli bacteria, then sacrificed at 30 min after injection, were used for later experiments. We found that mice treated with IPA presented markedly higher peritoneal macrophage phagocytosis than those treated with vehicle, but mice treated with CH223191 blunted the effect of IPA on peritoneal macrophage phagocytosis (**Figure 5B**). *In vitro*, THP-1 monocytes/macrophages were used to detect the effect of IPA on their phagocytosis. Likewise, we noted that although there was no statistical significance, IPA had a tendency to increase the phagocytosis of THP-1 cell (**Supplemental Figure 2**).

### Macrophages were critical in maintaining the survival protective effect of IPA on septic mice

Intraperitoneal administration with clodronate liposomes can deplete peritoneal macrophages, but will take longer to deplete macrophages in liver and spleen (ca. 3 days) (Sunderkötter et al., 2004). Likewise, we found that IPA treatment conspicuously increased the survival rate after CLP in mice. However, when macrophages were depleted from IPA pre-treated mice, mice in IPA group showed no increase in survival rate compared with vehicle group (**Figure 5C**). Therefore, we determined that macrophages are key to maintaining the improved survival seen in IPA treated mice after CLP.

### IPA levels in feces correlate with better outcomes in human sepsis

**Figure 6:**
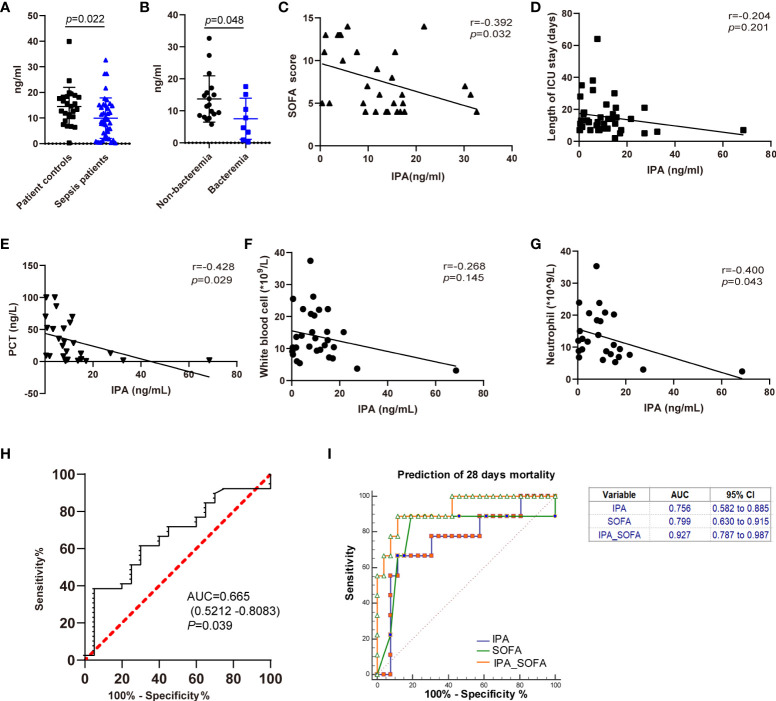
The associations between IPA in feces and outcomes of septic patients. **(A)** The level of IPA in feces from the studied patients. Data was shown as mean ± SD, n= 26 for patient controls, n= 41 for sepsis patients; unpaired t test. **(B)** The level of IPA in feces from the sepsis patients with or without bacteremia. Data was shown as mean ± SD, n= 18 for non-bacteremia patients, n= 8 for bacteremia patients; unpaired t test. **(C–G)** Correlation of IPA levels in feces with Sequential Organ Failure Assessment (SOFA) scores (**C**, n=30), length of ICU stay (**D**, n=41), procalcitonin (PCT) **(E,** n=26), white blood cells (**F**, n=31), and neutrophil **(G,** n=26) in the septic patients (Spearman’s rank correlation test). **(H)** Receiver operating characteristic curve (ROC) of IPA in feces for diagnosis of sepsis. Areas under the ROC curve for IPA, 0.665 (*p*=0.039). **(I)** Receiving operating characteristic (ROC) curve of IPA, SOFA score, and IPA in combination with SOFA score at admission for predicting 28-day mortality in septic patients.

To assess the associations between clinical implications and IPA, we first examined the level of IPA in feces of patients with sepsis. IPA levels in feces were significantly reduced in patients with sepsis compared with patient control subjects (**Figure 6A**). Septic patients with bacteremia had significantly lower IPA levels in feces when compared with septic patients without bacteremia (**Figure 6B**). In particular, reduced IPA was associated with worse clinical outcomes in patients with sepsis. Lower feces IPA levels were related to higher SOFA scores (**Figure 6C**), procalcitonin (**Figure 6E**), and neutrophils (**Figure 6G**) in the patients with sepsis on the day of ICU admission. Furthermore, there was a negative correlation between IPA and the length of ICU stay (**Figure 6D**), or white blood cells (**Figure 6F**) levels on the day of ICU admission.

### The clinical role of IPA in feces in diagnosing sepsis and predicting 28-day mortality

We further investigated the clinical role of IPA in feces in septic patients. The ROC curve of IPA in feces for the diagnosis of sepsis is shown in **Figure 6H**. The areas of ROC of IPA on day of ICU admission was 0.665 (p=0.039). SOFA score was used to predict the prognosis of septic patients (Li et al., 2020). Therefore, regarding the prediction of 28-day mortality, the area under the ROC curve for IPA on day of ICU admission was 0.756 (p=0.013, [95% CI] 0.582–0.885), and the AUC for SOFA score was 0.799 (p = 0.007, [95% CI] 0.630–0.915). Interestingly, there was no significant difference between IPA in feces and SOFA scores in predicting 28-day mortality in patients with sepsis (p=0.805). Moreover, the AUC of IPA in combination with SOFA score was 0.927 (p <0.0001, [95% CI] 0.787–0.987) (**Figure 6I**).

## Discussion

In present study, we first assessed the role of IPA in the pathophysiology of sepsis progression using a clinically relevant murine model of CLP. The main findings were summarized as follows: 1) IPA replenishment protected against mortality and alleviated vital organs injury in lethal sepsis mice; 2) The protective effect of IPA on septic mice depended on AhR; 3) AhR-dependent enhancement of IPA-induced macrophage phagocytosis contributed to protecting against sepsis. Furthermore, we also analyzed the associations between feces IPA and clinical implications in human patients with sepsis, and explored the role of IPA in diagnosis and prediction of 28-day mortality in patients with sepsis, summed up as the following major findings: 1) IPA levels in feces were significantly reduced in patients with sepsis compared with patient controls and septic patients with bacteremia had significantly lower IPA levels in feces compared with septic patients without bacteremia; 2) feces IPA levels were negatively related to the severity of sepsis, such as SOFA score, procalcitonin, neutrophils, the length of ICU stay, and white blood cells. 3) IPA in feces provided some diagnostic value in sepsis, and the efficacy of IPA and SOFA score in predicting 28-day mortality of sepsis was similar, and IPA increased the predictive ability of SOFA score.

Over the past 15 years, numerous studies have shown that gut microbiota can affect the risk of disease, including diabetes (Gurung et al., 2020; Mokhtari et al., 2021), heart disease (Tang et al., 2017; Deng et al., 2022), obesity (de Wouters d’Oplinter et al., 2021; Du et al., 2021), and depression (Simpson et al., 2021; Yuan et al., 2021). The important reason is that these microorganisms can produce a large number of bioactive molecules to circulate in various parts of the host. IPA is a product of tryptophan degradation by gut microbiota (Dodd et al., 2017). Although IPA has been reported to be a potential therapeutic agent for sepsis-induced gut microbiota disturbance (Fang et al., 2022), our study confirms the role of IPA in the progression of polymicrobial sepsis based on animal experiment and clinical data. In this study, we observed that IPA derived from gut microbiota was associated with the survival of septic mice, and IPA replenishment protected against CLP-induced mortality and alleviated vital organs injury in septic mice. Unexpectedly, compared with pretreatment, IPA post-treated septic mice did not show significant survival protection. This phenomenon could be attributed to the following possible reasons: 1) After IPA pretreatment, the host had a certain resistance to sepsis, but the post-treated mice lacked this process; 2) In our fatal (high-grade) sepsis model, the protective effects of IPA post-treatment might be masked by the effects of the disease itself. Therefore, a middle-grade sepsis model or a higher dose of IPA will be needed to further explore the effects of IPA post-treatment on septic mice.

Some studies have shown that IPA is a ligand for AhR and modulates astrocyte activity and central nervous system inflammation, and promotes human and murine intestinal homeostasis (Rothhammer et al., 2016; Sivaprakasam et al., 2017). In present study, consistent with previous studies, we found that AhR mediated the protective effect of IPA on septic mice. Enhanced phagocytosis and bacterial killing of macrophages are known for improving survival, decreasing bacterial burden, and attenuating tissue injury after sepsis. In our study, IPA rapidly enhanced the phagocytosis of E. coli by macrophages, but this effect was weakened by AhR inhibitor CH223191. However, compared with vehicle group, serum levels of IL-1β, IL-6 and TNF-α at 12 hours after CLP did not increase significantly not only in CLP group but also in IPA treatment group. For many years, a disproportionate inflammatory response to invasive infection was considered to be central to the pathogenesis of sepsis, but it is now clear that the host response is disturbed in a much more complex way, involving both sustained excessive inflammation and immune suppression. It may be argued that during sepsis there is not simply increased inflammation and/or immune suppression (inhibition and resolution of inflammation), but also no clear time demarcation between increased inflammation and immune suppression (Tom van der Poll et al., 2017). Previous study has shown that phagocytosis can induce proinflammatory cytokine production in murine macrophages (Acharya et al., 2019). This may explain why had no marked difference of proinflammatory cytokines among vehicle, CLP and IPA treated mice after CLP. Different mechanisms involved in phagocytosis of pathogens by macrophages include the interaction of specific receptors on the surface of the macrophages with ligands on the surface of the pathogens, the polymerization of actin, the internalization of phagocytic particle, and the formation of the mature phagolysosome by a series of fusion and fission events (Aderem and Underhill, 1999). Thus, further research is needed on how IPA regulates macrophage phagocytosis. Moreover, we found that the protective effects of IPA on septic mice could be rescued by depletion of macrophages with clodronate liposomes. These results are consistent with other studies on the role of macrophages in sepsis (Dahdah et al., 2014; Yang et al., 2019; Yan et al., 2020). Whether the transfer of macrophages from IPA-treated mice to different mice shows that the protection of IPA for septic mice needs further research.

The ultimate goal of animal research on sepsis is for clinical transformation. Here, we explore the relationship between IPA and sepsis in patients. First, we found that, on the day of ICU admission (day 0), feces IPA levels were significantly lower in septic patients when compared with patient controls. Then, there was a negative correlation between feces IPA levels and the severity of sepsis, including SOFA score, PCT, documented bacteremia, and so on. Moreover, the ROC curve of IPA in feces for the diagnosis of sepsis showed IPA levels in feces had a certain potential in sepsis diagnosis. In addition, the ROC curve of IPA in feces for prediction 28-day mortality of sepsis showed IPA had similar prediction ability with SOFA score, and increased the predictive ability of SOFA score. These findings suggest that IPA levels in feces may have a protective effect and can be used as one of the combined factors in diagnosis and prediction 28-day mortality in patients with sepsis.

Several limitations in this study should be considered. First, it is unclear if IPA is having a direct contribution, or if it is a marker of larger dysbiosis that contributes to sepsis outcomes. We only used a pharmacological antagonist of the AhR but AhR-deficient mice in present study. Then, exact mechanism of the decrease of IPA in feces after sepsis and how IPA regulates macrophage phagocytosis need further study. In addition, the clinical relationship between fecal IPA levels and sepsis resulted from the study on a limited number of patients. However, sepsis resulted in a significant decrease of fecal IPA in both human sepsis and murine sepsis, suggesting that the clinical value of fecal IPA in human patients with sepsis should be validated in a prospective clinical study with large samples and multiple centers.

## Conclusions

Collectively, our study suggests a protective role for gut microbiota-derived IPA during sepsis in both human and murine models. IPA is a protective factor for sepsis through improving survival and host control of infection by partially promoting aryl hydrocarbon receptor-dependent macrophages phagocytosis. our findings, therefore, not only reveal a previously unrecognized role of IPA in sepsis, but also suggest a potential strategy against sepsis.

## Data availability statement

The raw data supporting the conclusions of this article will be made available by the authors, without undue reservation.

## Ethics statement

The studies involving human participants were reviewed and approved by Research Ethics Board of Fujian Provincial Hospital, Fuzhou, China (Approval number: K2021-02-005). The patients/participants provided their written informed consent to participate in this study. The animal study was reviewed and approved by Animal Care and Use Committee of Southern Medical University, Guangzhou, China.

## Author contributions

Z-BH and K-XL conceived and designed the project. Z-BH and ZH performed all animal experiments and analyzed all animal data. YC, Z-PZ and C-XL performed all clinal experiments. S-DL, J-JH and F-LZ performed histology, macrophage phagocytosis and ELISA experiments. Z-BH and K-XL wrote the paper with the assistance of the other authors. All authors contributed to the article and approved the submitted version.

## Funding

This work was supported by the grants from the National Natural Science Foundation of China (No. 81730058 and 82172141 to K-XL) and the Foundation for Dean of Nanfang Hospital of Southern Medical University (No. 2019Z022 to K-XL).

## Acknowledgments

We thank Peng Chen for technical assistance.

## Conflict of interest

The authors declare that the research was conducted in the absence of any commercial or financial relationships that could be construed as a potential conflict of interest.

## Publisher’s note

All claims expressed in this article are solely those of the authors and do not necessarily represent those of their affiliated organizations, or those of the publisher, the editors and the reviewers. Any product that may be evaluated in this article, or claim that may be made by its manufacturer, is not guaranteed or endorsed by the publisher.

## References

[B1] Acharya D. Li X. R. L. Heineman R. E. Harrison R. E. (2019). Complement receptor-mediated phagocytosis induces proinflammatory cytokine production in murine macrophages. Front. Immunol. 10, 3049. doi: 10.3389/fimmu.2019.03049 31993058 PMC6970972

[B2] Aderem A. Underhill D. M. (1999). Mechanisms of phagocytosis in macrophages. Annu. Rev. Immunol. 17, 593–623. doi: 10.1146/annurev.immunol.17.1.593 10358769

[B3] Agudelo-Ochoa G. M. Valdés-Duque B. E. Giraldo-Giraldo N. A. Jaillier-Ramírez A. M. Giraldo-Villa A. Acevedo-Castaño I. . (2020). Gut microbiota profiles in critically ill patients, potential biomarkers and risk variables for sepsis. Gut Microbes 12 (1), 1707610. doi: 10.1080/19490976.2019.1707610 31924126 PMC7524144

[B4] Alexeev E. E. Lanis J. M. Kao D. J. Campbell E. L. Kelly C. J. Battista K. D. . (2018). Microbiota-derived indole metabolites promote human and murine intestinal homeostasis through regulation of interleukin-10 receptor. Am. J. pathol 188 (5), 1183–1194. doi: 10.1016/j.ajpath.2018.01.011 29454749 PMC5906738

[B5] Bendheim P. E. Poeggeler B. Neria E. Ziv V. Pappolla M. A. Chain D. G. (2002). Development of indole-3-propionic acid (OXIGON) for alzheimer’s disease. J. Mol. Neurosci. 19 (1-2), 213–217. doi: 10.1007/s12031-002-0036-0 12212784

[B6] Cecconi M. Evans L. Levy M. Rhodes A. (2018). Sepsis and septic shock. Lancet 392 (10141), 75–87. doi: 10.1016/S0140-6736(18)30696-2 29937192

[B7] Dahdah A. Gautier G. Attout T. Fiore F. Lebourdais E. Msallam R. . (2014). Mast cells aggravate sepsis by inhibiting peritoneal macrophage phagocytosis. J. Clin. Invest. 124 (10), 4577–4589. doi: 10.1172/JCI75212 25180604 PMC4191002

[B8] Delano M. J. Ward P. A. (2016). Sepsis-induced immune dysfunction: Can immune therapies reduce mortality? J. Clin. Invest. 126 (1), 23–31. doi: 10.1172/JCI82224 26727230 PMC4701539

[B9] Deng F. Hu J. J. Yang X. Sun Q. S. Lin Z. B. Zhao B. C. . (2021). Gut microbial metabolite pravastatin attenuates intestinal Ischemia/Reperfusion injury through promoting IL-13 release from type II innate lymphoid cells *via* IL-33/ST2 signaling. Front. Immunol. 12, 704836. doi: 10.3389/fimmu.2021.704836 34650552 PMC8505964

[B10] Deng F. Zhang L. Q. Wu H. Chen Y. Yu W. Q. Han R. H. . (2022). Propionate alleviates myocardial ischemia-reperfusion injury aggravated by angiotensin II dependent on caveolin-1/ACE2 axis through GPR41. Int. J. Biol. Sci. 18 (2), 858–872. doi: 10.7150/ijbs.67724 35002530 PMC8741842

[B11] Deng F. Zhao B. C. Yang X. Lin Z. B. Sun Q. S. Wang Y. F. . (2021). The gut microbiota metabolite capsiate promotes Gpx4 expression by activating TRPV1 to inhibit intestinal ischemia reperfusion-induced ferroptosis. Gut Microbes 13 (1), 1–21. doi: 10.1080/19490976.2021.1902719 PMC8009132 33779497

[B12] de Wouters d’Oplinter A. Rastelli M. Van Hul M. Delzenne N. M. Cani P. D. Everard A. (2021). Gut microbes participate in food preference alterations during obesity. Gut Microbes 13 (1), 1959242. doi: 10.1080/19490976.2021.1959242 34424831 PMC8386729

[B13] Dodd D. Spitzer M. H. Van Treuren W. Merrill B. D. Hryckowian A. J. Higginbottom S. K. . (2017). A gut bacterial pathway metabolizes aromatic amino acids into nine circulating metabolites. Nature. 551 (7682), 648–652. doi: 10.1038/nature24661 29168502 PMC5850949

[B14] Du J. Zhang P. Luo J. Shen L. Zhang S. Gu H. . (2021). Dietary betaine prevents obesity through gut microbiota-drived microRNA-378a family. Gut Microbes 13 (1), 1–19. doi: 10.1080/19490976.2020.1862612 PMC7889173 33550882

[B15] Fang H. Fang M. Wang Y. Zhang H. Li J. Chen J. . (2022). Indole-3-Propionic acid as a potential therapeutic agent for sepsis-induced gut microbiota disturbance. Microbiol. spectrum. 10 (3), e0012522. doi: 10.1128/spectrum.00125-22 PMC9241804 35658593

[B16] Fleischmann C. Scherag A. Adhikari N. K. Hartog C. S. Tsaganos T. Schlattmann P. . (2016). Assessment of global incidence and mortality of hospital-treated sepsis. Curr. Estimates Limitations. Am. J. Respir. Crit. Care Med. 193 (3), 259–272. doi: 10.1164/rccm.201504-0781OC 26414292

[B17] Gurung M. Li Z. You H. Rodrigues R. Jump D. B. Morgun A. . (2020). Role of gut microbiota in type 2 diabetes pathophysiology. EBioMedicine. 51, 102590. doi: 10.1016/j.ebiom.2019.11.051 31901868 PMC6948163

[B18] Gutiérrez-Vázquez C. Quintana F. J. (2018). Regulation of the immune response by the aryl hydrocarbon receptor. Immunity. 48 (1), 19–33. doi: 10.1016/j.immuni.2017.12.012 29343438 PMC5777317

[B19] Hotchkiss R. S. Monneret G. Payen D. (2013). Sepsis-induced immunosuppression: from cellular dysfunctions to immunotherapy. Nat. Rev. Immunol. 13 (12), 862–874. doi: 10.1038/nri3552 24232462 PMC4077177

[B20] Huang X. Venet F. Wang Y. L. Lepape A. Yuan Z. Chen Y. . (2009). PD-1 expression by macrophages plays a pathologic role in altering microbial clearance and the innate inflammatory response to sepsis. Proc. Natl. Acad. Sci. United States America. 106 (15), 6303–6308. doi: 10.1073/pnas.0809422106 PMC2669369 19332785

[B21] Hu J. Deng F. Zhao B. Lin Z. Sun Q. Yang X. . (2022). Lactobacillus murinus alleviate intestinal ischemia/reperfusion injury through promoting the release of interleukin-10 from M2 macrophages *via* toll-like receptor 2 signaling. Microbiome. 10 (1), 38. doi: 10.1186/s40168-022-01227-w 35241180 PMC8896269

[B22] Jin Z. Zhu Z. Liu S. Hou Y. Tang M. Zhu P. . (2020). TRIM59 protects mice from sepsis by regulating inflammation and phagocytosis in macrophages. Front. Immunol. 11, 263. doi: 10.3389/fimmu.2020.00263 32133014 PMC7041419

[B23] Konopelski P. Mogilnicka I. (2022). Biological effects of indole-3-Propionic acid, a gut microbiota-derived metabolite, and its precursor tryptophan in mammals’ health and disease. Int. J. Mol. Sci. 23 (3), 1222. doi:10.3390/ijms23031222 35163143 10.3390/ijms23031222 PMC8835432

[B24] Liu Z. Dai X. Zhang H. Shi R. Hui Y. Jin X. . (2020). Gut microbiota mediates intermittent-fasting alleviation of diabetes-induced cognitive impairment. Nat. Commun. 11 (1), 855. doi: 10.1038/s41467-020-14676-4 32071312 PMC7029019

[B25] Liu C. Huang S. Wu Z. Li T. Li N. Zhang B. . (2021). Cohousing-mediated microbiota transfer from milk bioactive components-dosed mice ameliorate colitis by remodeling colonic mucus barrier and lamina propria macrophages. Gut Microbes 13 (1), 1–23. doi: 10.1080/19490976.2021.1903826 PMC8018355 33789528

[B26] Li Y. Yan C. Gan Z. Xi X. Tan Z. Li J. . (2020). Prognostic values of SOFA score, qSOFA score, and LODS score for patients with sepsis. Ann. palliative Med. 9 (3), 1037–1044. doi: 10.21037/apm-20-984 32498525

[B27] Mokhtari P. Metos J. Anandh Babu P. V. (2021). Impact of type 1 diabetes on the composition and functional potential of gut microbiome in children and adolescents: possible mechanisms, current knowledge, and challenges. Gut Microbes 13 (1), 1–18. doi: 10.1080/19490976.2021.1926841 PMC8205092 34101547

[B28] Negatu D. A. Gengenbacher M. Dartois V. Dick T. (2020). Indole propionic acid, an unusual antibiotic produced by the gut microbiota, with anti-inflammatory and antioxidant properties. Front. Microbiol. 11, 575586. doi: 10.3389/fmicb.2020.575586 33193190 PMC7652848

[B29] Niu M. Chen P. (2021). Crosstalk between gut microbiota and sepsis. Burns Trauma 9, tkab036. doi: 10.1093/burnst/tkab036 34712743 PMC8547143

[B30] Ozdulger A. Cinel I. Koksel O. Cinel L. Avlan D. Unlu A. . (2003). The protective effect of n-acetylcysteine on apoptotic lung injury in cecal ligation and puncture-induced sepsis model. Shock (Augusta Ga). 19 (4), 366–372. doi: 10.1097/00024382-200304000-00012 12688549

[B31] Rittirsch D. Huber-Lang M. S. Flierl M. A. Ward P. A. (2009). Immunodesign of experimental sepsis by cecal ligation and puncture. Nat. Protoc. 4 (1), 31–36. doi: 10.1038/nprot.2008.214 19131954 PMC2754226

[B32] Rodriguez A. E. Ducker G. S. Billingham L. K. Martinez C. A. Mainolfi N. Suri V. . (2019). Serine metabolism supports macrophage IL-1β production. Cell Metab. 29 (4), 1003–11.e4. doi: 10.1016/j.cmet.2019.01.014 30773464 PMC6447453

[B33] Rothhammer V. Mascanfroni I. D. Bunse L. Takenaka M. C. Kenison J. E. Mayo L. . (2016). Type I interferons and microbial metabolites of tryptophan modulate astrocyte activity and central nervous system inflammation *via* the aryl hydrocarbon receptor. Nat. Med. 22 (6), 586–597. doi: 10.1038/nm.4106 27158906 PMC4899206

[B34] Sekine H. Mimura J. Oshima M. Okawa H. Kanno J. Igarashi K. . (2009). Hypersensitivity of aryl hydrocarbon receptor-deficient mice to lipopolysaccharide-induced septic shock. Mol. Cell. Biol. 29 (24), 6391–6400. doi: 10.1128/MCB.00337-09 19822660 PMC2786870

[B35] Shinde R. McGaha T. L. (2018). The aryl hydrocarbon receptor: Connecting immunity to the microenvironment. Trends Immunol. 39 (12), 1005–1020. doi: 10.1016/j.it.2018.10.010 30409559 PMC7182078

[B36] Simpson C. A. Diaz-Arteche C. Eliby D. Schwartz O. S. Simmons J. G. Cowan C. S. M. (2021). The gut microbiota in anxiety and depression - a systematic review. Clin. Psychol. review. 83, 101943. doi: 10.1016/j.cpr.2020.101943 33271426

[B37] Singer M. Deutschman C. S. Seymour C. W. Shankar-Hari M. Annane D. Bauer M. . (2016). The third international consensus definitions for sepsis and septic shock (Sepsis-3). Jama. 315 (8), 801–810. doi: 10.1001/jama.2016.0287 26903338 PMC4968574

[B38] Sivaprakasam S. Bhutia Y. D. Ramachandran S. Ganapathy V. (2017). Cell-surface and nuclear receptors in the colon as targets for bacterial metabolites and its relevance to colon health. Nutrients. 9 (8), 856. doi: 10.3390/nu9080856 PMC5579649 28796169

[B39] Sunderkötter C. Nikolic T. Dillon M. J. Van Rooijen N. Stehling M. Drevets D. A. . (2004). Subpopulations of mouse blood monocytes differ in maturation stage and inflammatory response. J. Immunol. 172 (7), 4410–4417. doi: 10.4049/jimmunol.172.7.4410 15034056

[B40] Suzuki S. Toledo-Pereyra L. H. Rodriguez F. J. Cejalvo D. (1993). Neutrophil infiltration as an important factor in liver ischemia and reperfusion injury. Modulating effects FK506 cyclosporine. Transplantation. 55 (6), 1265–1272. doi: 10.1097/00007890-199306000-00011 7685932

[B41] Tang W. H. Kitai T. Hazen S. L. (2017). Gut microbiota in cardiovascular health and disease. Circ. Res. 120 (7), 1183–1196. doi: 10.1161/CIRCRESAHA.117.309715 28360349 PMC5390330

[B42] Tom van der Poll T. van de Veerdonk F. L. Scicluna B. P. Netea M. G. (2017). The immunopathology of sepsis and potential therapeutic targets. Nat. Rev. Immunol. 17 (7), 407–420. doi: 10.1038/nri.2017.36 28436424

[B43] Venkatesh M. Mukherjee S. Wang H. Li H. Sun K. Benechet A. P. . (2014). Symbiotic bacterial metabolites regulate gastrointestinal barrier function *via* the xenobiotic sensor PXR and toll-like receptor 4. Immunity. 41 (2), 296–310. doi: 10.1016/j.immuni.2014.06.014 25065623 PMC4142105

[B44] Volkova M. Palmeri M. Russell K. S. Russell R. R. (2011). Activation of the aryl hydrocarbon receptor by doxorubicin mediates cytoprotective effects in the heart. Cardiovasc. Res. 90 (2), 305–314. doi: 10.1093/cvr/cvr007 21233252 PMC3078799

[B45] Wang L. Gong Z. Zhang X. Zhu F. Liu Y. Jin C. . (2020). Gut microbial bile acid metabolite skews macrophage polarization and contributes to high-fat diet-induced colonic inflammation. Gut Microbes 12 (1), 1–20. doi: 10.1080/19490976.2020.1819155 PMC7553752 33006494

[B46] Wiersinga W. J. Leopold S. J. Cranendonk D. R. van der Poll T. (2014). Host innate immune responses to sepsis. Virulence. 5 (1), 36–44. doi: 10.4161/viru.25436 23774844 PMC3916381

[B47] Xiao H. W. Cui M. Li Y. Dong J. L. Zhang S. Q. Zhu C. C. . (2020). Gut microbiota-derived indole 3-propionic acid protects against radiation toxicity *via* retaining acyl-CoA-binding protein. Microbiome. 8 (1), 69. doi: 10.1186/s40168-020-00845-6 32434586 PMC7241002

[B48] Yang X. Yin Y. Yan X. Yu Z. Liu Y. Cao J. (2019). Flagellin attenuates experimental sepsis in a macrophage-dependent manner. Crit. Care (London England). 23 (1), 106. doi: 10.1186/s13054-019-2408-7 PMC6446324 30944018

[B49] Yan X. Tu H. Liu Y. Chen T. Cao J. (2020). Interleukin-17D aggravates sepsis by inhibiting macrophage phagocytosis. Crit. Care Med. 48 (1), e58–e65. doi: 10.1097/CCM.0000000000004070 31634237

[B50] Yan J. Tung H. C. Li S. Niu Y. Garbacz W. G. Lu P. . (2019). Aryl hydrocarbon receptor signaling prevents activation of hepatic stellate cells and liver fibrogenesis in mice. Gastroenterology. 157 (3), 793–806.e14. doi: 10.1053/j.gastro.2019.05.066 31170413 PMC6707837

[B51] Yao R. Q. Ren C. Wang J. N. Wu G. S. Zhu X. M. Xia Z. F. . (2020). Publication trends of research on sepsis and host immune response during 1999-2019: A 20-year bibliometric analysis. Int. J. Biol. Sci. 16 (1), 27–37. doi: 10.7150/ijbs.37496 31892843 PMC6930382

[B52] Yuan X. Chen B. Duan Z. Xia Z. Ding Y. Chen T. . (2021). Depression and anxiety in patients with active ulcerative colitis: Crosstalk of gut microbiota, metabolomics and proteomics. Gut Microbes 13 (1), 1987779. doi: 10.1080/19490976.2021.1987779 34806521 PMC8632339

[B53] Zhao Z. H. Xin F. Z. Xue Y. Hu Z. Han Y. Ma F. . (2019). Indole-3-propionic acid inhibits gut dysbiosis and endotoxin leakage to attenuate steatohepatitis in rats. Exp. Mol. Med. 51 (9), 1–14. doi: 10.1038/s12276-019-0304-5 PMC6802644 31506421

[B54] Zhu J. Luo L. Tian L. Yin S. Ma X. Cheng S. . (2018). Aryl hydrocarbon receptor promotes IL-10 expression in inflammatory macrophages through src-STAT3 signaling pathway. Front. Immunol. 9, 2033. doi: 10.3389/fimmu.2018.02033 30283437 PMC6156150

