# Supplementary figures and images for: Gut microbiota-derived indole 3-propionic acid partially activates aryl hydrocarbon receptor to promote macrophage phagocytosis and attenuate septic injury
Source: Front Cell Infect Microbiol. 2022 Oct 10;12:1015386. doi: 10.3389/fcimb.2022.1015386 (PMC9589056; doi:10.3389/fcimb.2022.1015386)

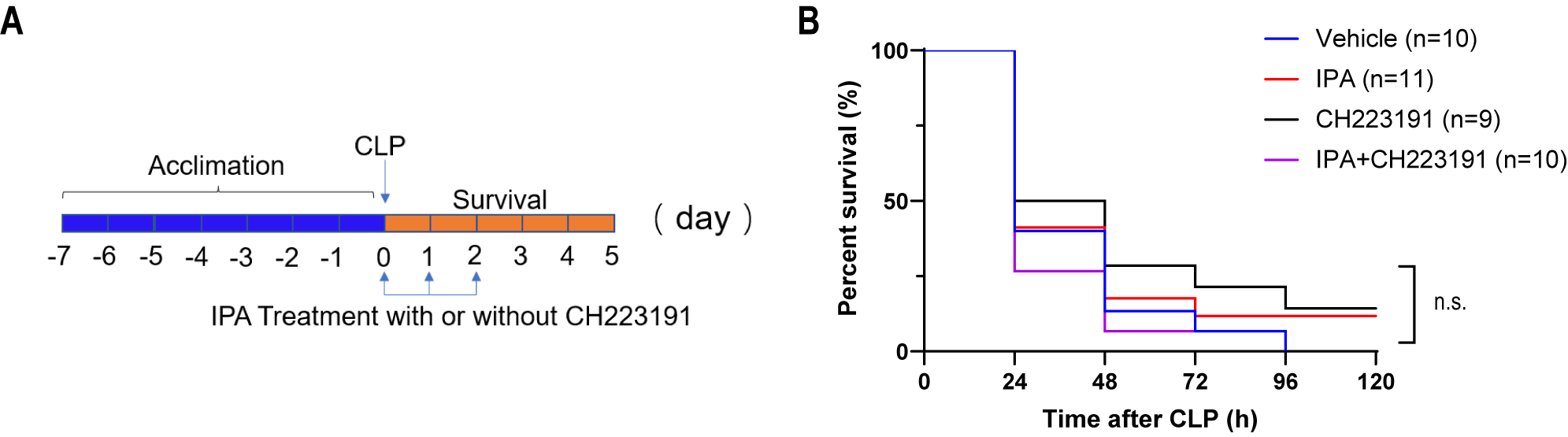

Supplement: Supplementary file 1 [file Image_1.tif]

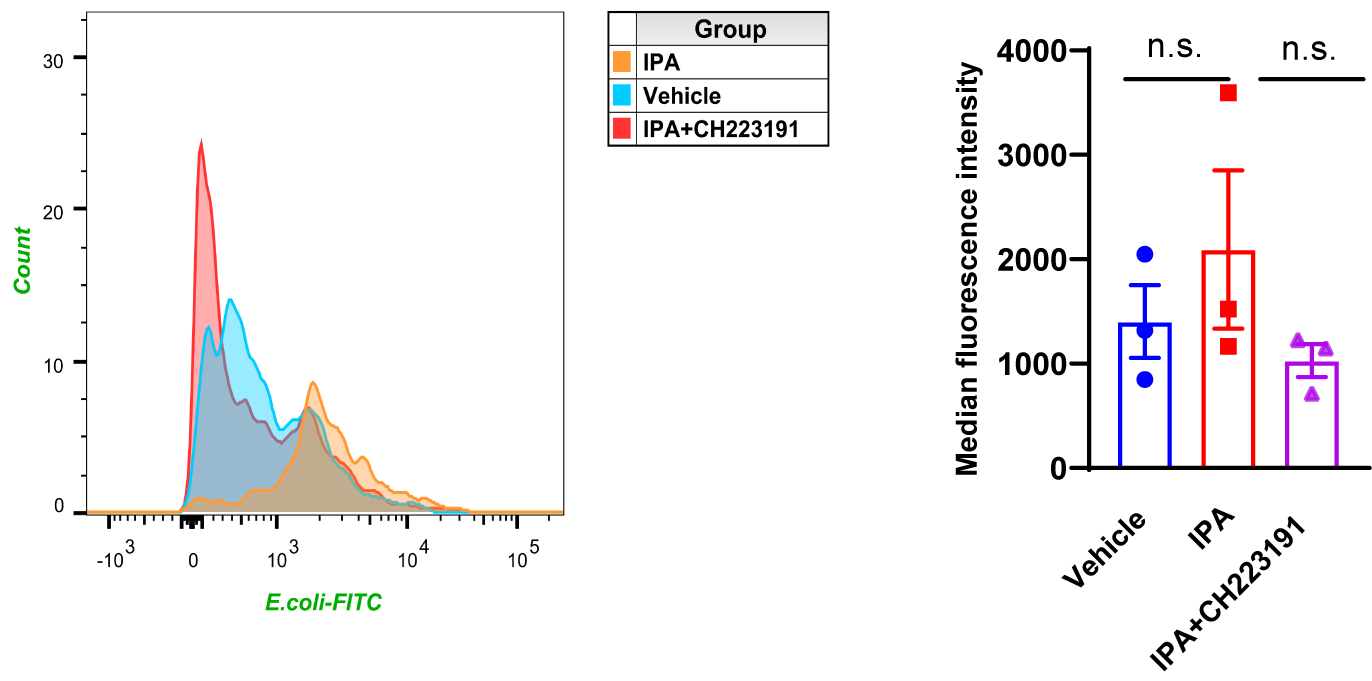

Supplement: Supplementary file 2 [file Image_2.tif]
